# Serum ω-3 Polyunsaturated Fatty Acids and Potential Influence Factors in Elderly Patients with Multiple Cardiovascular Risk Factors

**DOI:** 10.1038/s41598-018-19193-5

**Published:** 2018-01-18

**Authors:** Wenwen Liu, Xiaochuan Xie, Meilin Liu, Jingwei Zhang, Wenyi Liang, Xiahuan Chen

**Affiliations:** Department of Geriatrics, Peking University First Hospital, Beijing, 100034 China

## Abstract

Recent clinical trials failed to demonstrate that ω-3 polyunsaturated fatty acid (PUFA) supplement reduced cardiovascular events, which contradicted previous evidence. However, serum ω-3 PUFA concentrations of participants remained unclear in those studies. We aimed to investigate the definite relationship between serum concentrations of ω-3 PUFAs and coronary artery disease (CAD), and to explore the potential influence factors of ω-3 PUFAs. We selected Chinese in-patients (n = 460) with multiple cardiovascular risk factors or an established diagnosis of CAD. Serum ω-3 PUFAs, including eicosapentaenoic acid (EPA) and docosahexaenoic acid (DHA), were measured by liquid chromatography mass spectrometry. Serum concentrations of ω-3 PUFAs in CAD patients were lower than that in patients with cardiovascular risk factors. Furthermore, high serum DHA concentration was an independent protective factor of CAD after adjustment for confounding factors (OR: 0.52, p = 0.014). Alcohol intake (p = 0.036) and proton pump inhibitor (PPI) usage (p = 0.027) were associated with a decreased serum ω-3 PUFA concentration. We conclude that serum concentrations of ω-3 PUFAs may associate with a decreased CAD proportion, and DHA may serve as a protective factor of CAD. Serum ω-3 PUFA concentrations may be reduced by alcohol intake and certain drugs like PPIs.

## Introduction

ω-3 Polyunsaturated fatty acids (PUFAs), especially eicosapentaenoic acid (EPA) and docosahexaenoic acid (DHA), are known to have several beneficial effects, such as lowing blood lipids, alleviating immune injuries, inhibiting thrombogenesis, improving cognitive function, relieving depression, and restraining tumor growth^1–5^. Additionally, previous observational studies have shown that intake of ω-3 PUFAs may prevent the occurrence and progression of multiple cardiovascular diseases^6–8^.

Recently, the effectiveness of long-term intervention of ω-3 PUFAs as a secondary prevention of atherosclerotic diseases, including coronary artery disease (CAD), has been investigated in clinical trials. It seems that whether serum ω-3 PUFAs are associated with protection against these diseases remained controversial nowadays^9–11^. However, those intervention trials failed to measure serum ω-3 PUFA concentrations of participants. Moreover, large scale data on ω-3 PUFA concentrations are extremely lacking.

ω-3 PUFA intake is commonly measured by dietary methods, such as semi-quantitative food-frequency questionnaire (FFQ)^12,13^, and serum concentrations are determined by gas chromatography^14–18^. However, these two methods are not infallible. Recalling and reporting bias may influence the reliability of semi-quantitative FFQ. Gas chromatography, on the other hand, requires a time-consuming preparation process, which limits study sample size. LCMS is a highly sensitive analytical chemistry technique that is commonly employed to identify chemicals of particular masses in the presence of other chemicals (i.e. identify pure substances from mixtures of chemical intermediates)^19^.

Therefore, we conducted this study to estimate baseline serum concentrations of ω-3 PUFAs by LCMS, investigate define association of serum ω-3 PUFA concentrations with CAD and further analyze possible influence factors of serum ω-3 PUFAs in Chinese elderly.

## Methods

### Design and subjects

This was a cross-sectional, population-based study. Eligible participants were male and female in-patients with multiple cardiovascular risk factors or an established diagnosis of coronary artery disease (CAD), recruited between January 2015 and March 2016 from Peking University First Hospital. The definition of multiple cardiovascular risk factors included the following criterions (the first criterion was fundamental and at least combined with one of other criterions): 1) an age of 55 years or older; 2) hypertension (before drug treatment, seated systolic blood pressure ≥140 mmHg or seated diastolic blood pressure ≥90 mmHg or use of antihypertensive treatment); 3) hypercholesterolemia (total cholesterol ≥ 5.2 mmol/L/200 mg/dL or low density lipoprotein ≥ 3.4 mmol/L/130 mg/dL) or non-high density lipoprotein ≥4.1 mmol/L/160 mg/dL or triglyceride ≥ 1.7 mmol/L/150 mg/dL or use of lipid-lowering treatment); 4) diabetes mellitus (fasting blood glucose ≥ 7.0 mmol/l or blood glucose ≥ 11.1 mmol/L after oral glucose tolerance test within 2 hours or use of antidiabetics); 5) status as a current smoker; 6) obesity [a body-mass index (the weight in kilograms divided by the square of the height in meters) of 28 or more]; and 7) or a family history of premature cardiovascular disease (cardiovascular disease at <55 years of age in male immediate family or at <65 years of age in in female immediate family). A diagnosis of CAD was patients with at least 50% stenosis by area in at least one coronary artery confirmed by coronary computed tomography angiography (CTA) or coronary angiography (CAG). Exclusion criteria included: 1) age less than 55 years old; 2) failure to obtain informed consent; 3) death during hospitalization; 4) current treatment with ω-3 PUFAs; and 5) a diagnosis of acute coronary syndrome (ACS) within 3 months prior to enrollment. Finally, a total of 460 elderly subjects (CAD: 190, CAD-Risk: 270; mean age: 69.07 ± 12.03; male/female: 336/124) were selected for this study. The detailed characteristics of the study participants are summarized in Table 1.

**Table 1:** Main characteristics of participants and the comparison between patients with cardiovascular risk factors and established diagnosis of CAD.

Variables	All participants (n = 460)	CAD-Risk (n = 270)	CAD (n = 190)	P value
Age (y)	69.07 ± 12.03	68.60 ± 12.10	69.74 ± 11.92	0.319
Male [n (%)]	336(73.0%)	197(73.0%)	139(73.2%)	0.963
Smoking habit/Current [n (%)]	55(18.0%)	27(17.9%)	28(17.9%)	0.988
Alcoholic habit/Current [n (%)]	49(16.0%)	24(15.9%)	25(16.0%)	0.975
Hypertension [n (%)]	306(66.5%)	164(60.7%)	142(74.7%)	0.002
Hyperlipidemia [n (%)]	237(51.5%)	127(47.0%)	110(57.9%)	0.022
Diabetes mellitus [n (%)]	146(31.7%)	73(27.0%)	73(38.4%)	0.010
BMI (Kg/m^2^)	25.12 ± 3.33	24.90 ± 3.27	25.43 ± 3.40	0.097
SBP (mmHg)	133.79 ± 19.23	131.98 ± 17.96	136.32 ± 20.63	0.017
DBP (mmHg)	77.15 ± 11.39	77.78 ± 10.96	76.25 ± 11.93	0.157
Blood test results				
EPA (μg/L)	381.00	412.00	326.51	0.002
IQR	247.76–702.44	265.76–713.55	233.57–652.92	
DHA (μg/L)	1480.69	1645.30	1159.90	0.013
IQR	752.79–3130.73	884.35–3203.07	638.00–3068.10	
EPA/DHA	0.26	0.25	0.28	0.321
IQR	0.14–0.45	0.15–0.43	0.12–0.51	
Glucose (mmol/L)	5.74 ± 1.80	5.43 ± 1.33	6.19 ± 2.24	<0.001
HbA1c (%)	6.34 ± 1.23	6.13 ± 0.94	6.60 ± 1.47	<0.001
HCY (umol/L)	13.02	13.15	13.00	1.000
IQR	10.53–16.50	10.90–16.14	10.10–16.57	
TG (mmol/L)	1.30	1.39	1.21	0.008
IQR	0.93–1.83	0.97–1.89	0.89–1.67	
TC (mmol/L)	4.21 ± 1.15	4.49 ± 1.19	3.81 ± 0.95	<0.001
HDL-C (mmol/L)	1.10 ± 0.30	1.13 ± 0.32	1.04 ± 0.26	0.002
LDL-C (mmol/L)	2.50 ± 0.89	2.71 ± 0.87	2.21 ± 0.83	<0.001
WBC (10^9^/L)	6.47 ± 2.30	6.19 ± 1.95	6.86 ± 2.67	0.003
hsCRP (mg/L)	1.74	1.74	1.73	1.000
IQR	0.54–7.31	0.60–5.93	0.51–9.01	
Medication [n (%)]				
Aspirin	138(45.1%)	23(15.1%)	115(74.7%)	<0.001
Clopidogrel	105(34.3%)	7(4.6%)	98(63.6%)	<0.001
Statins	186(60.8%)	49(32.2%)	137(89.0%)	<0.001
ACEIs	41(13.4%)	12(7.9%)	29(18.8%)	0.005
ARBs	80(26.1%)	38(25.0%)	42(27.3%)	0.651
β-Blockers	157(51.3%)	45(29.6%)	112(72.7%)	<0.001
CCBs	104(34.0%)	48(31.6%)	56(36.4%)	0.377
Diuretics	66(21.6%)	31(20.4%)	35(22.7%)	0.620
Nitrates	92(30.1%)	13(8.6%)	79(51.3%)	<0.001
Antidiabetics	83(27.1%)	39(25.7%)	44(28.5%)	0.543
PPIs	95(31.0%)	31(20.4%)	64(41.6%)	<0.001

SBP, seated systolic blood pressure; DBP, seated diastolic blood pressure; TG, triglyceride; TC, total cholesterol; HDL-C, high-density lipoprotein; LDL-C, low density lipoprotein; Glucose, fasting blood glucose; HbA1c, hemoglobin A1C; HCY, homocysteine; WBC, white blood cells; hsCRP, high sensitive C-reactive protein; ACEIs, angiotensin converting enzyme inhibitors; ARBs, angiotensin receptor blocking agents; CCBs, calcium channel blockers; PPIs, proton pump inhibitors. IQR, inter quartile range.

This study was performed in accordance with the ethical principles of the Declaration of Helsinki. The study protocol was approved by our hospital Institutional Ethics Committee (PUFH 2015-1008) and written informed consent was obtained from all participants.

### Measurement of Serum ω-3 PUFAs

Fasting serum samples were collected early in the morning after the participants had fasted for at least 12 hours overnight, and stored at −80 °C until analyses. Serum ω-3 PUFAs, including EPA and DHA, were measured by liquid chromatography mass spectrometry (LCMS)^19^ (Fan-Xing Biological Technology - Beijing Co., Ltd).

**Figure 1:**
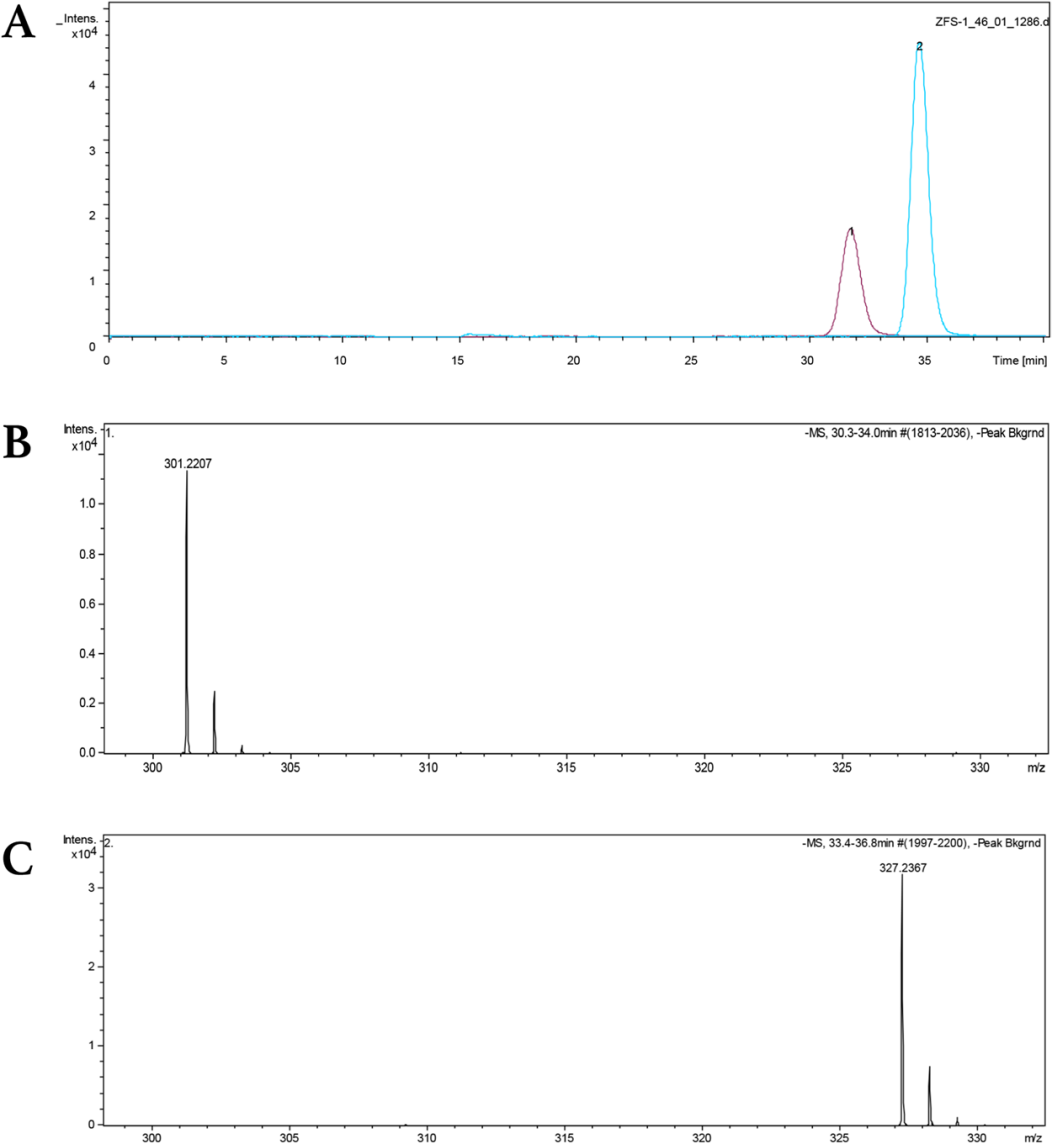
The measurement of serum ω-3 PUFA concentrations with liquid chromatography mass spectrometry (LCMS). (**A**) ω-3 PUFA chromatogram. EPA peak was labelled 1, and DHA peak was labelled 2. (**B**) EPA mass spectrogram. ω-3 PUFAs were detected in positive ion mode which made the detected mass was 1 less than actual molecule. EPA molecular mass: 302.35. (**C**) DHA mass spectrogram. EPA molecular mass: 328.48.

10 μL-serum samples were separated by a chromatographic column (BEH C18, 5 μm × 4.6 × 150 mm, Aglient) and eluted with a mobile phase of 40% A (water containing 5 mmol/L ammonium acetate) and 60% B (acetonitrile containing 5 mmol/L ammonium acetate) at a flow rate of 0.1 mL/min. The MS detection (MicroQ-TOFII, Bruker Dalton) was performed with electrospray ionization in the positive ion mode with multiple reaction monitors (Fig. 1). Drying temperature was 250 °C.

Peak intensity was linearly correlated with ω-3 PUFA concentration (R^2^ > 0.99). Therefore, serum concentrations of EPA and DHA were calculated according to the equation of linear regression between peak intensity and concentration.

EPA peak intensity = 3005.6 × EPA concentration (μg/L)/200 − 2070.3; R^2^ = 0.9994

DHA peak intensity = 2224.2 × DHA concentration (μg/L)/200 + 1082.7; R^2^ = 0.9941

In addition, other biochemical parameters, including fasting plasma glucose, hemoglobin A1C, homocysteine, triglycerides, total cholesterol, high-density lipoprotein, low density lipoprotein, white blood cells, red blood cells, hemoglobin, mean corpuscular haemoglobin concentration, high sensitive C-reactive protein, Vitamin D, alanine aminotransferase, albumin, plasma creatinine, potassium, sodium and brain natriuretic peptide were measured by standard analytical methods with routine laboratory testing at our hospital. The estimated glomerular filtration rate (eGFR) was calculated according to the formula: eGFR (ml/min per 1.73 m^2^) = 175 × Pcr^−1.234^ × age^−0.179^ × 0.79 (if female)^20^.

### Statistical analyses

The Shapiro-Wilk test was used to determine the distribution normality of each continuous variable. Normally distributed, continuous variables were presented as mean ± SD, and any differences between the two groups were tested by the student t-test. Non-normally distributed, continuous variables were presented as median with inter quartile range (IQR), and the Kruskal-Wallis test was used to discern differences between the two groups. Categorical variables were presented as percentages, and potential differences between two groups were estimated with the chi-square test or Fisher’s exact test. Possible relationships between two variables were analyzed by the Pearson test. Binary logistic regression analyses were conducted to estimate the correlationship between ω-3 PUFAs and CAD, and linear regression analyses were used to trace the possible influence factors of ω-3 PUFAs. P values less than 0.05 were regarded as statistically significant. All data analyses were performed using SPSS 20.0 software (Statistical Package for the Social Sciences, SPSS Ins., Chicago, IL).

### Data Availability

The datasets generated during and analysed during the current study are available from the corresponding author on reasonable request.

## Results

### Study participants

Finally, 460 in-patients met the inclusion criteria in the cross-sectional study. Clinical characteristics of study participants are shown in Table 1. For all the participants, serum EPA concentration ranged from 25.30 to 4275.57 μg/L (median, interquartile: 381.00, 247.76/702.44 μg/L), and serum DHA concentration ranged from 31.68 to 25307.69 μg/L (median, interquartile: 1480.69, 752.79/3130.73 μg/L). There were no significant differences in age, gender, smoking/alcoholic habits, or body mass index (BMI) between the CAD group and CAD-Risk group. Nevertheless, the proportions of subjects with hypertension (74.7 vs. 60.7%, p = 0.002), hyperlipidemia (57.9 vs. 47.0%, p = 0.022) and diabetes mellitus (38.4 vs. 27.0%, p = 0.010) were higher in the CAD group compared with that in the CAD-Risk group. Serum lipid concentrations in the CAD group were lower than that in the CAD-Risk group (median triglyceride/TG: 1.21 vs. 1.39 mmol/L, p = 0.008; mean total cholesterol/TC: 3.81 vs. 4.49 mmol/L, p < 0.001; mean high density lipoprotein cholesterol/HDL-C: 1.04 vs. 1.13 mmol/L, p = 0.002; mean low density lipoprotein cholesterol/LDL-C: 2.21 vs. 2.71 mmol/L, p < 0.001). In addition, the concentrations of fasting blood glucose (mean 6.19 vs. 5.43 mmol/L, p < 0.001) and hemoglobin A1C (HbA1c) (mean 6.60 vs. 6.13%, p < 0.001) were higher in the CAD group than the CAD-Risk group. Moreover, we found that the CAD group took more drugs than the CAD-Risk group [aspirin (74.7 vs. 15.1%, p < 0.001); clopidogrel (63.6 vs. 4.6%, p < 0.001); statins (89.0 vs. 32.2%, p < 0.001); angiotensin converting enzyme inhibitors/ACEIs (18.8 vs. 7.9%, p = 0.005); β-Blockers (72.7 vs. 29.6%, p < 0.001); nitrates (51.3 vs. 8.6%, p < 0.001); proton pump inhibitors/PPIs (41.6 vs. 20.4%, p < 0.001)]. Finally, serum concentrations of ω-3 PUFAs in CAD patients were significantly lower than that in CAD-Risk patients (median EPA: 326.51 vs. 412.00 μg/L, p = 0.002; median DHA: 1159.90 vs. 1645.30 μg/L, p = 0.013).

**Figure 2:**
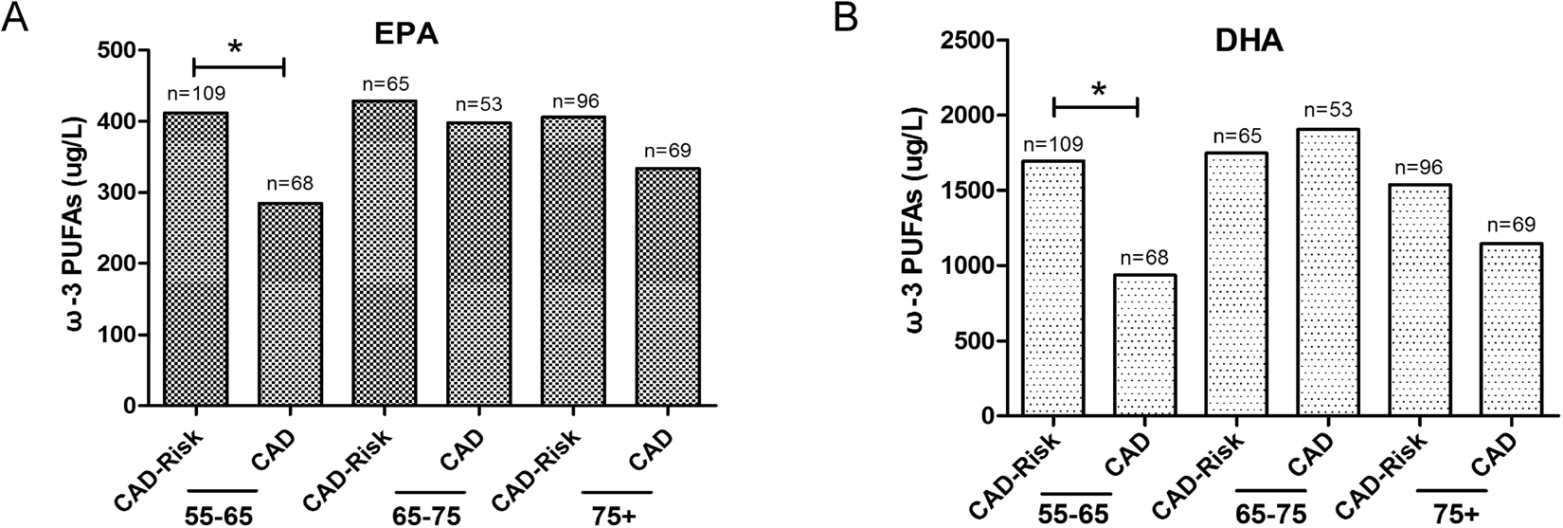
Serum ω-3 PUFA concentrations in age subgroups. (**A**) Serum EPA concentrations in age subgroups. 55–65: 55 ≤ age ≤ 65, 65–75: 65 < age ≤ 75, 75+: age > 75. Serum EPA concentrations in CAD patients were significant lower than that in non-CAD patients in 55–65 subgroup. (**B**) Serum DHA concentrations in age subgroups. CAD patients had significantly lower serum DHA concentration than non-CAD patients in 55–65 subgroup.

We further allocated the participants into three subgroups (55–65: 55 ≤ age ≤ 65, 65–75: 65 < age ≤ 75, 75 + : age > 75) according to their age (Fig. 2A,B). In 55–65 subgroup, both EPA and DHA concentrations in CAD patients were significantly lower than that in CAD-Risk patients (median EPA: 284.46 vs. 412.00 μg/L, p = 0.002; median DHA: 938.83 vs. 1694.72 μg/L, p = 0.028). There were no significant differences in serum concentrations of ω-3 PUFAs between the CAD group and CAD-Risk group in 65–75 subgroup (median EPA: 397.88 vs. 429.02 μg/L, p = 0.976; median DHA: 1906.06 vs. 1746.81 μg/L, p = 0.930) or 75+ subgroup (median EPA: 333.34 vs. 406.00 μg/L, p = 0.319; median DHA: 1145.52 vs. 1538.34 μg/L, p = 0.148).

### Identification of independent influence factors of CAD

In order to figure out the independent influence factors of CAD, we set the binary variable “cardiovascular disease” as the dependent variable, CAD-Risk as “0” and CAD as “1”. Totally 11 covariates were set as the independent variables, and passed through the binary logistic regression model using the Backward-Wald method. The 11 covariates were age, gender, smoking habit, alcohol intake, BMI, hypertension, hyperlipidemia, fasting blood glucose, white blood cells, EPA_G (0 = EPA ≤ median 381.00 μg/L, 1 = EPA > median 381.00 μg/L) and DHA_G (0 = DHA ≤ median 1480.69 μg/L, 1 = DHA > median 1480.69 μg/L). Eventually, four covariates were entered the equation, shown in Table 2. The variables included high serum DHA concentration (OR: 0.52; 95% CI: 0.31, 0.88; p = 0.014), female (OR: 0.54; 95% CI: 0.314, 0.913; p = 0.022), hyperlipidemia (OR: 2.67, 95% CI: 1.59, 4.50; p < 0.001), and fasting blood glucose concentration (OR: 1.19; 95% CI: 1.03, 1.38; p = 0.020).

**Table 2:** Independent risk factors of CAD.

Covariates	B	S.E.	Wald	df	OR	95% CI for OR	P value
Female	−0.63	0.27	5.27	1	0.54	0.31, 0.91	0.022
Hyperlipidemia	0.98	0.27	13.68	1	2.67	1.58, 4.50	<0.001
DHA_G	−0.65	0.27	6.02	1	0.52	0.31, 0.88	0.014
Glucose	0.18	0.08	5.39	1	1.19	1.03, 1.38	0.020

Binary logistic regression analysis. The dependent variable was CAD (CAD-Risk as “0”, CAD as “1”). The 11 covariates were age, gender (0 = male, 1 = female), smoking habit (0 = never smoke or quit smoking more than one year, 1 = still smoking), alcohol intake (0 = no alcohol intake or quit using alcohol more than one month, 1 = still drinking), BMI, hypertension (0 = without hypertension, 1 = with hypertension), hyperlipidemia (0 = without hyperlipidemia, 1 = with hyperlipidemia), fasting blood glucose, white blood cells, EPA_G (0 = EPA ≤ median 381.00 μg/L, 1 = EPA > median 381.00 μg/L), and DHA_G (0 = DHA ≤ median 1480.69 μg/L, 1 = DHA > median 1480.69 μg/L). B, partial regression coefficient; S.E., standard error for B; df, degree of freedom; OR, odds ratio; CI, confidence interval.

### Baseline characteristics of participants according to ω-3 PUFAs concentrations

We further allocated the participants into different groups according to their median serum EPA and DHA concentrations to analyze the relationship between ω-3 PUFA concentrations and CAD. As shown in Table 3, no significant differences were found in age, gender, smoking/alcoholic habits, or BMI between the two groups (high EPA group vs. low EPA group; high DHA group vs. low DHA group). The CAD proportion was lower in the high EPA group compared with  the low EPA group (34.0 vs. 48.7%, p < 0.001), and similar result was found in the high DHA and low DHA groups (35.1 vs. 46.9%, p = 0.010). All serum lipid concentrations were found to be higher in the high DHA group compared with the low DHA group (median TG: 1.41 vs. 1.21 mmol/L, p = 0.005; mean TC: 4.38 vs. 4.06 mmol/L, p = 0.003; mean HDL-C: 1.13 vs. 1.06 mmol/L, p = 0.029; mean LDL-C: 2.61 vs. 2.40 mmol/L, p = 0.012). Similarly, the median TG (1.41 vs. 1.21 mmol/L, p = 0.001) and mean TC (4.34 vs. 4.08 mmol/L, p = 0.016) concentrations were higher in the high EPA group compared with the low EPA group. Certain blood biochemical markers, including albumin (ALB), red blood cells (RBC), hemoglobin (Hb) and potassium (K) were significantly higher in the high ω-3 PUFA groups than the low ω-3 PUFA groups (p values < 0.05). However, other biochemical markers, such as creatine kinase (CK) and brain natriuretic peptide (BNP) were significantly lower in the high ω-3PUFA groups than the low ω-3PUFA groups (p values < 0.05). In addition, the high EPA group took less ACEIs (8.2 vs. 17.4%, p = 0.019) and diuretics (14.9 vs. 24.7%, p = 0.013) than the low EPA group; while the high DHA group used less diuretics (16.5 vs. 26.1%, p = 0.045) and PPIs (25.2 vs. 35.8%, p = 0.047) than the low DHA group.

**Table 3:** Comparison between different concentrations of serum ω-3 PUFAs.

Variables	EPA groups			DHA groups		
	Low EPA n = 230	High EPA n = 229	P value	Low DHA n = 228	High DHA n = 228	P value
Age (y)	68.95 ± 12.73	69.14 ± 11.31	0.868	69.35 ± 12.05	68.70 ± 12.00	0.567
Male [n (%)]	164(71.3%)	171(74.7%)	0.400	168(73.7%)	165(72.4%)	0.752
Smoking habit/Current [n (%)]	34(19.4%)	21(15.9%)	0.413	33(19.8%)	22(15.9%)	0.388
Alcoholic habit/Current [n (%)]	36(20.6%)	13(9.8%)	0.011	31(18.6%)	17(12.3%)	0.136
Hypertension [n (%)]	150(65.2%)	155(67.7%)	0.576	153(67.1%)	151(66.2%)	0.843
Hyperlipidemia [n (%)]	114(49.6%)	122(53.3%)	0.454	109(47.8%)	126(55.3%)	0.122
Diabetes mellitus [n (%)]	71(30.9%)	74(32.3%)	0.763	78(34.2%)	68(29.8%)	0.315
BMI (Kg/m2)	25.04 ± 3.49	25.19 ± 3.18	0.631	25.28 ± 3.46	24.96 ± 3.22	0.321
Cardiovascular disease						
CAD [n (%)]	112(48.7%)	78(34.1%)	0.001	107(46.9%)	80(35.1%)	0.010
PCI/CABG	58(56.3%)	45(43.7%)	0.421	58(56.9%)	44(43.1%)	0.914
Coronary restenosis [n (%)]	45(48.4%)	22(38.6%)	0.242	44(48.9%)	21(36.2%)	0.129
Blood test						
EPA (μg/L)	250.88	702.44	<0.001	286.62	685.87	<0.001
IQR	183.278–311.289	492.47–991.18		202.04–387.43	381.26–984.93	
DHA (μg/L)	810.75	3032.40	<0.001	753.74	3129.60	<0.001
IQR	508.70–1463.41	1480.69–5846.94		492.30–1042.32	2220.42–5878.16	
EPA/DHA	0.29	0.25	0.133	0.44	0.17	<0.001
IQR	0.12–0.52	0.15–0.39		0.28–0.66	0.11–0.25	
VitD (nmol/L)	44.52 ± 14.29	49.97 ± 13.10	0.006	47.50 ± 15.07	47.86 ± 13.01	0.857
TG (mmol/L)	1.21	1.41	0.001	1.21	1.41	0.005
IQR	0.87–1.70	1.06–1.95		0.88–1.64	1.06–2.01	
TC (mmol/L)	4.08 ± 1.09	4.34 ± 1.19	0.016	4.06 ± 1.23	4.38 ± 1.04	0.003
HDL-C (mmol/L)	1.07 ± 0.29	1.12 ± 0.31	0.089	1.06 ± 0.28	1.13 ± 0.32	0.029
LDL-C (mmol/L)	2.43 ± 0.85	2.57 ± 0.92	0.105	2.40 ± 0.88	2.61 ± 0.89	0.012
ALT (IU/L)	18.00	19.00	0.540	18.00	18.00	0.854
IQR	13.00–25.00	14.00–26.00		14.00–25.00	14.00–26.00	
ALB (g/L)	40.62 ± 5.33	42.25 ± 6.14	0.003	40.42 ± 6.18	42.42 ± 5.25	<0.001
RBC (10^12^/L)	4.36 ± 0.64	4.48 ± 0.61	0.035	4.38 ± 0.64	4.47 ± 0.62	0.140
Hb (g/L)	135.75 ± 18.93	140.65 ± 18.40	0.005	136.30 ± 19.10	140.06 ± 18.47	0.034
MCHC (g/L)	340.47 ± 16.20	344.48 ± 24.47	0.040	341.52 ± 16.47	343.38 ± 24.37	0.342
Pcr (µmol/L)	105.98 ± 72.06	96.97 ± 44.53	0.108	102.56 ± 66.23	100.46 ± 53.61	0.709
eGFR (mL/min/1.73 m2)	73.88 ± 26.86	75.06 ± 19.11	0.591	75.73 ± 27.08	73.18 ± 18.78	0.243
K (mmol/L)	3.96 ± 0.49	4.06 ± 0.50	0.022	3.96 ± 0.53	4.06 ± 0.46	0.043
Na (mmol/L)	141.86 ± 18.27	141.14 ± 2.91	0.558	142.02 ± 18.32	140.98 ± 3.07	0.403
GLU (mmol/L)	5.82 ± 2.04	5.66 ± 1.52	0.329	5.76 ± 1.53	5.73 ± 2.05	0.855
CK (IU/L)	74.00	68.00	0.368	81.00	63.00	0.008
IQR	49.00–115.00	50.00–93.00		52.50–115.50	45.00–92.00	
BNP (pg/ml)	118.00	74.00	0.093	120.00	66.00	0.004
IQR	41.75–340.75	31.25–246.00		44.50–377.75	29.50–210.50	
HsCRP (mg/L)	1.64	2.03	0.610	1.91	1.52	0.799
IQR	0.60–6.27	0.50–10.24		0.60–8.88	0.50–4.64	
Medication [n (%)]						
Aspirin	85(49.4%)	53(39.6%)	0.095	81(49.1%)	55(39.6%)	0.096
Clopidogrel	60(34.9%)	45(33.6%)	0.784	61(37.0%)	42(30.2%)	0.201
Statins	107(62.2%)	79(59.0%)	0.563	108(65.5%)	76(54.7%)	0.055
ACEIs	30(17.4%)	11(8.2%)	0.019	26(15.8%)	14(10.1%)	0.144
ARBs	41(23.8%)	39(29.1%)	0.258	40(24.2%)	40(28.8%)	0.371
β-Blockers	95(55.2%)	62(46.3%)	0.131	92(55.8%)	64(46.0%)	0.091
CCBs	61(35.5%)	43(32.1%)	0.512	52(31.7%)	51(36.7%)	0.342
Diuretics	46(26.7%)	20(14.9%)	0.013	43(26.1%)	23(16.5%)	0.045
Nitrates	57(33.1%)	35(26.1%)	0.173	56(34.1%)	36(25.9%)	0.128
Antidiabetics	47(27.3%)	36(26.8%)	0.776	45(27.3%)	38(27.3%)	0.652
PPIs	52(30.2%)	43(32.1%)	0.753	59(35.8%)	35(25.2%)	0.047

PCI/CABG, percutaneous coronary intervention/coronary artery bypass grafting; Coronary restenosis, coronary restenosis confirmed by CTA or CAG within one year; VitD, Vitamin D; ALT, alanine aminotransferase; ALB, albumin; TBIL, total bilirubin; RBC, red blood cells; Hb, hemoglobin; MCHC, mean corpuscular haemoglobin concentration; Pcr, plasma creatinine; eGFR, estimated glomerular filtration rate; K, potassium; Na, sodium; GLU, fasting blood glucose; CK, creatine kinase; BNP, brain natriuretic peptide.

### Identification of influence factors of ω-3 PUFAs

Continuous variable “EPA” and “DHA” were set as the dependent variable (y) in order to identify possible influence factors of ω-3 PUFAs. Totally 16 covariates were set as the independent variables (x), and passed through the linear regression model with Stepwise method to select the independent correlated covariates of serum ω-3 PUFA concentrations. Finally, two covariates, DHA_G (95% CI: 517.58, 826.36; p < 0.001) and alcohol intake (95% CI: −398.54, −13.18; p = 0.036) entered the EPA equation (R^2^ = 0.29) (see Table 4). Two covariates, EPA_G (95% CI: 4701.01, 6629.07; p < 0.001) and PPIs (95% CI: −2164.66, −130.81; p = 0.027) entered the DHA equation (see Table 5).

**Table 4:** Correlation analysis of serum concentration of EPA.

Model	B(unstandardized)	S.E.	Beta(standardized)	t	95% CI for B	P value
(Constant)	299.53	53.53	—	5.60	193.95, 405.12	<0.001
DHA_G	671.97	78.27	0.52	8.59	517.58, 826.36	<0.001
Alcohol intake	−205.86	97.69	−0.13	−2.11	−398.543, −13.18	0.036

Linear regression analysis. The continuous variable “EPA” was set as the dependent variable (y). The 16 covariates were age, gender, smoking habit, alcohol intake, BMI, hypertension, diabetes mellitus, RBC, Hb, ALB, DHA_G, K, ACEIs (0 = no take, 1 = take), CCBs (0 = no take, 1 = take), Statins (0 = no take, 1 = take), and PPIs (0 = no take, 1 = take). B, unstandardized partial regression coefficient; S.E., standard error for B; Beta, standardized partial regression coefficient; t, t-test value. R^2^ = 0.288.

**Table 5:** Correlation analysis of serum concentration of DHA.

Model	B(unstandardized)	S.E.	Beta(standardized)	t	95% CI for B	P value
(Constant)	1424.35	2330.88	—	0.61	−3173.54, 6022.24	0.542
EPA_G	5665.04	488.71	0.63	11.59	4701.01, 6629.07	<0.001
PPIs	−1147.74	515.53	−0.12	−2.23	−2164.66, −130.81	0.027

Statistical analysis: linear regression analysis. The continuous variable “DHA” was set as the dependent variable (y). The 16 covariates were age, gender, smoking habit, alcohol intake, BMI, hypertension, diabetes mellitus, RBC, Hb, ALB, EPA_G, K, ACEIs, CCBs, Statins, and PPIs. R^2^ = 0.432.

## Discussion

To date, whether ω-3 PUFAs are associated with a reduced risk of cardiovascular events remain controversial, and data on serum ω-3 PUFA concentrations are still lacking worldwide. Therefore, we performed this study to elucidate the potential relationship between serum ω-3 PUFA concentrations and CAD in the Chinese elderly. The comparison between patients with cardiovascular risk factors and established diagnosis of CAD demonstrated that serum concentrations of ω-3 PUFAs, including EPA and DHA, were lower in patients with CAD than those with cardiovascular risk factors. Furthermore, the concentration differences between CAD and CAD-Risk group were significant in 55–65 (55 ≤ age ≤ 65) subgroups. This may result from complicated combined diseases and drugs that the older patients have, which may affect the protective effect of ω-3 PUFAs on CAD.

The CAD proportion was significantly lower in the high ω-3 PUFA group compared with the low ω-3 PUFA group. Unfortunately, no significant differences were found in coronary restenosis or revascularization proportion between different ω-3 PUFA concentration groups (see Table 3), which suggest that ω-3 PUFA may benefit CAD occurrence, while its role in CAD progression is not outstanding. Regression analyses revealed that DHA could serve as a protective factor of CAD, after adjustment for age, gender and co-morbidity conditions (OR: 0.52, 95% CI: 0.31, 0.88; p = 0.014). Although, the Pearson correlation analysis showed serum concentrations of EPA and DHA were positively correlated with each other (r = 0.53, p < 0.001), similar protective effect against CAD was not observed for EPA. It is possible that the sample size in the current study may not be sufficient to detect the beneficial effects of EPA or the protective effect of EPA on CAD may be less than DHA.

ω-3 PUFAs are components of erythrocyte membranes and necessary fatty acids that must be obtained from food. Therefore, we valued several biomarkers associated with red blood cells (RBC, Hb and mean corpuscular haemoglobin concentration/MCHC) and nutrient status (BMI, Vitamin D, potassium, sodium and albumin). The analysis results corresponded to our knowledge that high density of red blood cells and good nutrient status were associated with high ω-3 PUFA concentrations^21,22^. Additionally, we estimated other potential influence factors, such as smoking/alcoholic habit, hepatic (alanine aminotransferase) and renal (plasma creatinine and eGFR) function (see Table 3). Certain factors were found to affect serum ω-3 PUFAs. For example, regular alcohol intake and PPI use were correlated with a decreased serum ω-3 PUFA concentrations after adjustment for confounding factors (see Tables 4 and 5). Alcohol and PPI intake may affect the metabolic absorption of ω-3 PUFA from diet, generating reduced serum ω-3 PUFA concentrations. These findings indicate that reducing alcohol and PPI intake may contribute to a high serum ω-3 PUFA concentration. Considering that it has not been reported before, the findings require further investigations to confirm.

Previous observational studies employed self-reported FFQ to estimate PUFA intake. Food conversion estimations are imprecise as ω-3 PUFA amounts vary by food source and cooking methods and. In addition, different metabolic capabilities of ω-3 PUFAs contribute to altered ω-3 PUFA levels in different populations. Blood-based biomarkers of ω-3 PUFA intake are more objective and more accurate estimates of biological exposure^23^. Only a small number of studies have measured fatty acids, with many of these studies limited by sample size. According to our data, serum ω-3 PUFA concentrations were lower than that in other reports, which may suggest that the Chinese elderly, whose average daily intake of ω-3 PUFAs is relatively lower than that in Western and Japanese populations, may actually have lower baseline serum ω-3 PUFA concentrations. Besides, these results may be secondary to LCMS, a different yet more sensitive and precise measurement method. Additionally, the sample size of the study thus far is the largest that directly measures serum ω-3 PUFA concentrations.

Recent randomized controlled trials (RCTs) and meta-analyses fail to show that ω-3 PUFA intervention has beneficial effects in cardiovascular events^9–11^, which contradicts the findings of previous epidemiological studies. Potential reasons for this discrepancy may include the followings: First, the protective effects of ω-3 PUFAs against cardiovascular diseases may take many years to develop, and therefore the follow-up of recent RCTs may not be long enough. Second, doses of ω-3 PUFAs used by these studies (300–900 mg/day) were lower than the recommendation concentration (over 1000 mg/day)^24^, which may be insufficient to induce clinical benefits. Third, the biosynthesis of ω-3 PUFA is inefficient and varies from person to person^25–27^. We also found that serum lipid concentrations, including TG, TC, HDL-C, and LDL-C, in the CAD group were markedly lower than that in the CAD-Risk group, which may be attributed to the higher usage rate of statins (89.0 vs. 32.2%, p < 0.001, shown in Table 1) in CAD patients for secondary prevention. Long-term RCTs support the concept that combining statins with ω-3 fatty acids seems to further decrease CAD risk in primary prevention and CAD mortality in secondary prevention^28^. It is obvious that the cardiovascular events have been effectively retard with improved secondary prevention for cardiovascular diseases, which may weaken the protective effect of ω-3 PUFAs on CAD. Despite the inconsistent results obtained by previous studies, the American Heart Association (AHA) still recommend patients with cardiovascular diseases, especially those with CAD to take ω-3 PUFA supplements (IIa), and future multi-center studies with longer follow-up in different populations are needed to better elucidate the actual roles of ω-3 PUFAs in the prevention of cardiovascular diseases^29^.

There is no denying that some restrictions exist in this study. First, this was a cross-sectional study, which precluded us from obtaining a definite conclusion on the cause-effect relationship between ω-3 PUFAs and CAD. Second, in order to obtain the most comprehensive biochemical parameters for tracing potential influence factors of ω-3 PUFAs, the study design was hospital-based. Therefore, we could not rule out the possibility of selection bias. Third, considering that recalling and reporting bias may markedly influence the reliability of baseline ω-3 PUFA intake calculation, we didn’t rely on dietary patterns, which may also affect serum ω-3 PUFA concentrations. Larger-scale and long-term studies are still needed to confirm our findings.

## Conclusions

The current study suggests that high serum ω-3 PUFA concentration is associated with decreased CAD proportion at a relatively younger age. Moreover, DHA may be an independent protective factor of CAD. Additionally, serum ω-3 PUFA concentration may be reduced by alcohol intake and certain drugs like PPIs.
